# 17-(Pyrimidin-2-yl)-8,16-dioxa-17-aza­tetra­cyclo­[7.7.1.0^2,7^.0^10,15^]hepta­deca-2,4,6,10,12,14-hexa­ene

**DOI:** 10.1107/S1600536812000931

**Published:** 2012-01-18

**Authors:** M. Aslam, I. Anis, N. Afza, M. Ibrahim, S. Yousuf

**Affiliations:** a Pharmaceutical Research Centre, PCSIR Labs. Complex, Karachi, Pakistan; b Department of Chemistry, University of Karachi, Karachi, Pakistan; c H.E.J. Research Institute of Chemistry, International Center for Chemical and Biological Sciences, University of Karachi, Karachi 75270, Pakistan

## Abstract

In the title compound, C_18_H_13_N_3_O_2_, the benzene rings form a dihedral angle of 78.49 (9)°. The dihedral angles between the benzene rings and the pyrimidine ring are 76.53 (10) and 27.73 (11)°. The two *cis-*fused six-membered heterocyclic rings adopt half-chair confirmations. In the crystal, mol­ecules are linked by C—H⋯O hydrogen bonds, forming chains parallel to the *b* axis.

## Related literature

For the biological activity of Schiff bases, see: Khan *et al.* (2009 ▶). For the crystal structures of Schiff bases, see: Aslam *et al.* (2011 ▶); Zeb & Yousuf (2011 ▶). For the importance of carbon–nitro­gen bond-formation reactions for elucidating the mechanism of racemization and transamination reactions in biological systems, see: Lau *et al.* (1999 ▶). 
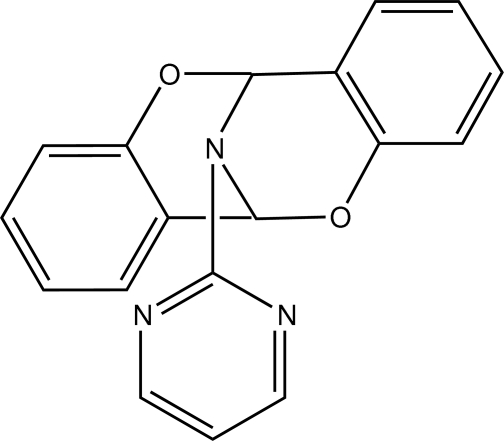


## Experimental

### 

#### Crystal data

C_18_H_13_N_3_O_2_
*M* *_r_* = 303.31
Monoclinic, 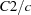

*a* = 30.004 (4) Å
*b* = 6.6083 (9) Å
*c* = 15.123 (2) Å
β = 99.652 (4)°
*V* = 2956.0 (7) Å^3^
*Z* = 8
Mo *K*α radiation
μ = 0.09 mm^−1^
*T* = 273 K
0.54 × 0.09 × 0.08 mm

#### Data collection

Bruker SMART APEX CCD area-detector diffractometer
Absorption correction: multi-scan (*SADABS*; Bruker, 2000 ▶) *T*_min_ = 0.952, *T*_max_ = 0.993
8329 measured reflections
2739 independent reflections
2026 reflections with *I* > 2σ(*I*)
*R*_int_ = 0.032

#### Refinement

*R*[*F* ^2^ > 2σ(*F* ^2^)] = 0.041
*wR*(*F* ^2^) = 0.097
*S* = 1.03
2739 reflections
208 parameters
H-atom parameters constrained
Δρ_max_ = 0.16 e Å^−3^
Δρ_min_ = −0.12 e Å^−3^

### 

Data collection: *SMART* (Bruker, 2000 ▶); cell refinement: *SAINT* (Bruker, 2000 ▶); data reduction: *SAINT*; program(s) used to solve structure: *SHELXS97* (Sheldrick, 2008 ▶); program(s) used to refine structure: *SHELXL97* (Sheldrick, 2008 ▶); molecular graphics: *SHELXTL* (Sheldrick, 2008 ▶); software used to prepare material for publication: *SHELXTL*, *PARST* (Nardelli, 1995 ▶) and *PLATON* (Spek, 2009 ▶).

## Supplementary Material

Crystal structure: contains datablock(s) global, I. DOI: 10.1107/S1600536812000931/pv2501sup1.cif

Structure factors: contains datablock(s) I. DOI: 10.1107/S1600536812000931/pv2501Isup2.hkl

Supplementary material file. DOI: 10.1107/S1600536812000931/pv2501Isup3.cml

Additional supplementary materials:  crystallographic information; 3D view; checkCIF report

## Figures and Tables

**Table 1:** Hydrogen-bond geometry (Å, °)

*D*—H⋯*A*	*D*—H	H⋯*A*	*D*⋯*A*	*D*—H⋯*A*
C5—H5*A*⋯O2^i^	0.93	2.48	3.199 (2)	134

Symmetry code: (i) 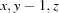.

**Fig. 1.**
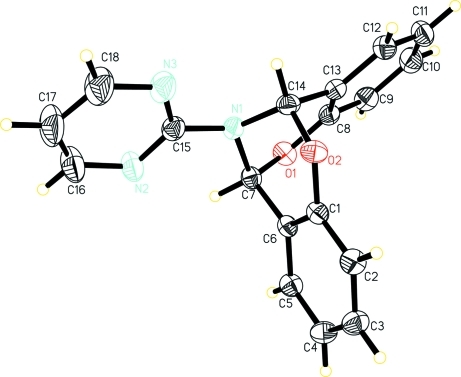
The molecular structure of the title molecule with displacement ellipsoids drawn at 30% probability level.

**Fig. 2.**
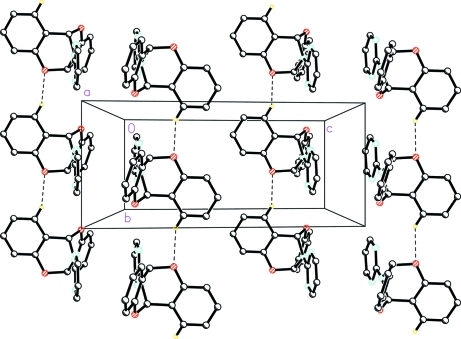
The crystal packing of the title compound showing intermolecular hydrogen bonds; the hydrogen atoms not involved in hydrogen bonding have been excluded for clarity.

## supplementary crystallographic information

### Comment

In organic chemistry, the reactions invloving the carbon-nitrogen bond formation are very important for elucidating the mechanism of racemisation and transamination reactions in biological systems (Lau *et al.*, 1999). The title compound was synthesized as a part of our on going research on Schiff base ligands (Khan *et al.*, 2009; Aslam *et al.*, 2011; Zeb & Yousuf, 2011).

The title compound (Fig. 1) is composed of five fused rings including two benzene (C1–C6 and C8–C13), one pyrimidine (N2/N3/C15–18) and two six membered heterocyclic rings (O1/N1/C7/C8/C13/C14 and O2/N1/C1/C6/C7/C14). The benzene (C8–C13) and pyrimidine (N2/N3/C15–C18) rings are twisted by 27.72 (10)° with respect to each other and form dihedral angles of 76.55 (10) and 78.48 (9)°, respectively, with the benzene ring (C1–C6). The cis fused six membered heterocyclic rings (O1/N1/C7/C8/C13/C14) and (O2/N1/C1/C6/C7/C14) adopt half-chair conformations; the atoms N1 and C7 lie -0.483 (3) and 0.311 (3) Å, respectively, from the plane defined by atoms (O1/C8/C13/C14) and the atoms N1 and C14 lie -0.456 (3) and 0.300 (3) Å, respectively, from the plane defined by atoms (O2/C1/C6/C7). In the crystal structure, the molecules are linked to form chains *via* C5—H5A···O2 intermolecular hydrogen bonds (Table 1) and lie parallel to the *b*-axix (Fig. 2). The bond lengths and angles in the title molecule are in accord with the corresponding bond lengths and angles reported in closely related structures (Aslam *et al.*, 2011; Zeb & Yousuf, 2011).

### Experimental

A mixture of salicylaldehyde (2 mol) and 2-aminopyrimidine (1 mol) in ethanol (50 ml) along with 3 drops of conc. H_2_SO_4_ was refluxed for 5 h at 343 K. The mixture was kept at room temperature for ten days to obtain light yellow crystals. The crystalline product was collected, washed with cooled ethanol and dried to afford the title compound in 80% yield. Recrystalization by slow evaporation of an ethanol solution of the title compound afforded light yellow crystals suitable for single-crystal X-ray diffraction studies. All chemicals were purchased from Sigma-Aldrich.

### Refinement

The aryl and methine H-atoms were positioned geometrically with C—H = 0.93 and 0.98 Å, respectively, and constrained to ride on their parent atoms with *U*_iso_(H) = 1.2*U*_eq_(C).

### Crystal data

**Table:**

C_18_H_13_N_3_O_2_	*F*(000) = 1264
*M**_r_* = 303.31	*D*_x_ = 1.363 Mg m^−^^3^
Monoclinic, *C*2/*c*	Mo *K*α radiation, λ = 0.71073 Å
Hall symbol: -C 2yc	Cell parameters from 1521 reflections
*a* = 30.004 (4) Å	θ = 2.7–21.4°
*b* = 6.6083 (9) Å	µ = 0.09 mm^−^^1^
*c* = 15.123 (2) Å	*T* = 273 K
β = 99.652 (4)°	Needle, yellow
*V* = 2956.0 (7) Å^3^	0.54 × 0.09 × 0.08 mm
*Z* = 8	

### Data collection

**Table:**

Bruker SMART APEX CCD area-detector diffractometer	2739 independent reflections
Radiation source: fine-focus sealed tube	2026 reflections with *I* > 2σ(*I*)
graphite	*R*_int_ = 0.032
ω scan	θ_max_ = 25.5°, θ_min_ = 1.4°
Absorption correction: multi-scan (*SADABS*; Bruker, 2000)	*h* = −36→35
*T*_min_ = 0.952, *T*_max_ = 0.993	*k* = −8→7
8329 measured reflections	*l* = −18→18

### Refinement

**Table:**

Refinement on *F*^2^	Primary atom site location: structure-invariant direct methods
Least-squares matrix: full	Secondary atom site location: difference Fourier map
*R*[*F*^2^ > 2σ(*F*^2^)] = 0.041	Hydrogen site location: inferred from neighbouring sites
*wR*(*F*^2^) = 0.097	H-atom parameters constrained
*S* = 1.03	*w* = 1/[σ^2^(*F*_o_^2^) + (0.0358*P*)^2^ + 1.0438*P*] where *P* = (*F*_o_^2^ + 2*F*_c_^2^)/3
2739 reflections	(Δ/σ)_max_ < 0.001
208 parameters	Δρ_max_ = 0.16 e Å^−^^3^
0 restraints	Δρ_min_ = −0.12 e Å^−^^3^

### Special details

**Table:**

Geometry. All e.s.d.'s (except the e.s.d. in the dihedral angle between two l.s. planes) are estimated using the full covariance matrix. The cell e.s.d.'s are taken into account individually in the estimation of e.s.d.'s in distances, angles and torsion angles; correlations between e.s.d.'s in cell parameters are only used when they are defined by crystal symmetry. An approximate (isotropic) treatment of cell e.s.d.'s is used for estimating e.s.d.'s involving l.s. planes.
Refinement. Refinement of *F*^2^ against ALL reflections. The weighted *R*-factor *wR* and goodness of fit *S* are based on *F*^2^, conventional *R*-factors *R* are based on *F*, with *F* set to zero for negative *F*^2^. The threshold expression of *F*^2^ > σ(*F*^2^) is used only for calculating *R*-factors(gt) *etc*. and is not relevant to the choice of reflections for refinement. *R*-factors based on *F*^2^ are statistically about twice as large as those based on *F*, and *R*- factors based on ALL data will be even larger.

### Fractional atomic coordinates and isotropic or equivalent isotropic displacement parameters (Å^2^)

**Table:**

	*x*	*y*	*z*	*U*_iso_*/*U*_eq_	
O1	0.14375 (4)	−0.35324 (17)	0.63616 (8)	0.0445 (3)	
O2	0.13833 (4)	0.06985 (16)	0.78989 (7)	0.0416 (3)	
N1	0.09854 (4)	−0.0699 (2)	0.65391 (9)	0.0406 (4)	
N2	0.02176 (5)	−0.1398 (3)	0.63059 (12)	0.0602 (5)	
N3	0.04887 (5)	0.2011 (3)	0.64007 (12)	0.0628 (5)	
C1	0.13535 (5)	−0.1148 (2)	0.83042 (11)	0.0355 (4)	
C2	0.14564 (6)	−0.1211 (3)	0.92321 (11)	0.0437 (4)	
H2A	0.1552	−0.0048	0.9555	0.052*	
C3	0.14156 (6)	−0.3003 (3)	0.96712 (12)	0.0494 (5)	
H3A	0.1485	−0.3050	1.0294	0.059*	
C4	0.12721 (6)	−0.4732 (3)	0.91988 (12)	0.0488 (5)	
H4A	0.1245	−0.5939	0.9502	0.059*	
C5	0.11688 (6)	−0.4670 (3)	0.82775 (12)	0.0414 (4)	
H5A	0.1070	−0.5837	0.7961	0.050*	
C6	0.12111 (5)	−0.2880 (2)	0.78159 (10)	0.0333 (4)	
C7	0.10824 (5)	−0.2779 (3)	0.68069 (11)	0.0380 (4)	
H7A	0.0810	−0.3595	0.6622	0.046*	
C8	0.18023 (5)	−0.2236 (3)	0.63998 (11)	0.0400 (4)	
C9	0.21881 (6)	−0.2982 (3)	0.61168 (12)	0.0504 (5)	
H9A	0.2197	−0.4308	0.5914	0.060*	
C10	0.25570 (6)	−0.1738 (4)	0.61407 (13)	0.0579 (5)	
H10A	0.2816	−0.2231	0.5954	0.070*	
C11	0.25481 (6)	0.0230 (4)	0.64381 (13)	0.0571 (5)	
H11A	0.2801	0.1056	0.6456	0.069*	
C12	0.21623 (6)	0.0969 (3)	0.67091 (12)	0.0483 (5)	
H12A	0.2156	0.2298	0.6910	0.058*	
C13	0.17824 (5)	−0.0248 (3)	0.66860 (11)	0.0382 (4)	
C14	0.13536 (5)	0.0582 (3)	0.69334 (11)	0.0399 (4)	
H14A	0.1303	0.1946	0.6682	0.048*	
C15	0.05409 (6)	0.0009 (3)	0.64170 (11)	0.0453 (5)	
C16	−0.02032 (7)	−0.0673 (4)	0.61509 (18)	0.0796 (7)	
H16A	−0.0442	−0.1587	0.6062	0.096*	
C17	−0.02996 (8)	0.1338 (5)	0.6117 (2)	0.0925 (9)	
H17A	−0.0596	0.1806	0.6009	0.111*	
C18	0.00588 (8)	0.2636 (4)	0.62481 (18)	0.0828 (8)	
H18A	0.0001	0.4020	0.6231	0.099*	

### Atomic displacement parameters (Å^2^)

**Table:**

	*U*^11^	*U*^22^	*U*^33^	*U*^12^	*U*^13^	*U*^23^
O1	0.0480 (7)	0.0438 (8)	0.0434 (7)	−0.0028 (6)	0.0124 (5)	−0.0111 (6)
O2	0.0520 (7)	0.0298 (7)	0.0446 (7)	−0.0027 (5)	0.0127 (5)	−0.0019 (5)
N1	0.0359 (8)	0.0398 (9)	0.0447 (8)	0.0015 (6)	0.0026 (6)	0.0035 (7)
N2	0.0370 (9)	0.0657 (12)	0.0771 (12)	−0.0010 (8)	0.0074 (8)	−0.0018 (9)
N3	0.0496 (10)	0.0545 (11)	0.0825 (13)	0.0138 (8)	0.0057 (9)	0.0044 (9)
C1	0.0333 (9)	0.0305 (10)	0.0441 (10)	0.0017 (7)	0.0103 (7)	−0.0010 (8)
C2	0.0492 (10)	0.0399 (11)	0.0425 (10)	−0.0030 (8)	0.0095 (8)	−0.0081 (8)
C3	0.0582 (11)	0.0500 (12)	0.0398 (10)	−0.0014 (9)	0.0075 (9)	0.0010 (9)
C4	0.0578 (12)	0.0384 (11)	0.0510 (12)	−0.0007 (9)	0.0112 (9)	0.0083 (9)
C5	0.0440 (10)	0.0312 (10)	0.0494 (11)	−0.0011 (8)	0.0087 (8)	−0.0027 (8)
C6	0.0299 (8)	0.0303 (9)	0.0405 (9)	0.0016 (7)	0.0082 (7)	−0.0031 (7)
C7	0.0352 (9)	0.0355 (10)	0.0432 (10)	−0.0013 (7)	0.0059 (7)	−0.0047 (8)
C8	0.0408 (9)	0.0479 (11)	0.0311 (9)	−0.0009 (8)	0.0055 (7)	−0.0001 (8)
C9	0.0533 (11)	0.0573 (13)	0.0422 (10)	0.0080 (10)	0.0130 (9)	−0.0030 (9)
C10	0.0432 (11)	0.0820 (17)	0.0509 (12)	0.0062 (11)	0.0145 (9)	0.0035 (11)
C11	0.0421 (11)	0.0755 (16)	0.0543 (12)	−0.0113 (10)	0.0095 (9)	0.0030 (11)
C12	0.0477 (11)	0.0517 (12)	0.0444 (11)	−0.0067 (9)	0.0043 (8)	0.0015 (9)
C13	0.0400 (9)	0.0405 (11)	0.0336 (9)	0.0007 (8)	0.0042 (7)	0.0036 (8)
C14	0.0450 (10)	0.0348 (10)	0.0398 (10)	−0.0011 (8)	0.0068 (8)	0.0050 (8)
C15	0.0422 (10)	0.0535 (13)	0.0405 (10)	0.0071 (9)	0.0081 (8)	0.0030 (9)
C16	0.0400 (12)	0.088 (2)	0.110 (2)	0.0023 (12)	0.0114 (12)	−0.0051 (15)
C17	0.0437 (13)	0.094 (2)	0.137 (2)	0.0221 (14)	0.0056 (14)	−0.0016 (18)
C18	0.0608 (15)	0.0690 (17)	0.116 (2)	0.0225 (13)	0.0080 (14)	0.0020 (14)

### Geometric parameters (Å, °)

**Table:**

O1—C8	1.383 (2)	C5—H5A	0.9300
O1—C7	1.4409 (18)	C6—C7	1.511 (2)
O2—C1	1.3749 (19)	C7—H7A	0.9800
O2—C14	1.4499 (19)	C8—C13	1.387 (2)
N1—C15	1.396 (2)	C8—C9	1.390 (2)
N1—C14	1.440 (2)	C9—C10	1.374 (3)
N1—C7	1.449 (2)	C9—H9A	0.9300
N2—C15	1.334 (2)	C10—C11	1.378 (3)
N2—C16	1.334 (3)	C10—H10A	0.9300
N3—C15	1.333 (2)	C11—C12	1.380 (3)
N3—C18	1.337 (2)	C11—H11A	0.9300
C1—C2	1.386 (2)	C12—C13	1.391 (2)
C1—C6	1.390 (2)	C12—H12A	0.9300
C2—C3	1.373 (2)	C13—C14	1.503 (2)
C2—H2A	0.9300	C14—H14A	0.9800
C3—C4	1.378 (3)	C16—C17	1.359 (3)
C3—H3A	0.9300	C16—H16A	0.9300
C4—C5	1.376 (2)	C17—C18	1.364 (3)
C4—H4A	0.9300	C17—H17A	0.9300
C5—C6	1.390 (2)	C18—H18A	0.9300
			
C8—O1—C7	114.10 (13)	C10—C9—C8	119.22 (19)
C1—O2—C14	113.85 (12)	C10—C9—H9A	120.4
C15—N1—C14	120.49 (15)	C8—C9—H9A	120.4
C15—N1—C7	119.85 (14)	C9—C10—C11	120.91 (18)
C14—N1—C7	109.76 (13)	C9—C10—H10A	119.5
C15—N2—C16	114.73 (19)	C11—C10—H10A	119.5
C15—N3—C18	114.63 (19)	C10—C11—C12	119.59 (18)
O2—C1—C2	117.26 (14)	C10—C11—H11A	120.2
O2—C1—C6	122.08 (14)	C12—C11—H11A	120.2
C2—C1—C6	120.62 (15)	C11—C12—C13	120.83 (19)
C3—C2—C1	119.53 (16)	C11—C12—H12A	119.6
C3—C2—H2A	120.2	C13—C12—H12A	119.6
C1—C2—H2A	120.2	C8—C13—C12	118.52 (16)
C2—C3—C4	120.66 (17)	C8—C13—C14	120.47 (15)
C2—C3—H3A	119.7	C12—C13—C14	120.97 (16)
C4—C3—H3A	119.7	N1—C14—O2	111.19 (13)
C5—C4—C3	119.87 (17)	N1—C14—C13	108.15 (14)
C5—C4—H4A	120.1	O2—C14—C13	111.07 (13)
C3—C4—H4A	120.1	N1—C14—H14A	108.8
C4—C5—C6	120.64 (16)	O2—C14—H14A	108.8
C4—C5—H5A	119.7	C13—C14—H14A	108.8
C6—C5—H5A	119.7	N3—C15—N2	127.55 (17)
C5—C6—C1	118.67 (15)	N3—C15—N1	116.23 (16)
C5—C6—C7	121.01 (15)	N2—C15—N1	116.20 (17)
C1—C6—C7	120.25 (14)	N2—C16—C17	123.2 (2)
O1—C7—N1	109.07 (13)	N2—C16—H16A	118.4
O1—C7—C6	111.90 (12)	C17—C16—H16A	118.4
N1—C7—C6	109.23 (13)	C16—C17—C18	116.9 (2)
O1—C7—H7A	108.9	C16—C17—H17A	121.6
N1—C7—H7A	108.9	C18—C17—H17A	121.6
C6—C7—H7A	108.9	N3—C18—C17	123.0 (2)
O1—C8—C13	121.61 (15)	N3—C18—H18A	118.5
O1—C8—C9	117.48 (16)	C17—C18—H18A	118.5
C13—C8—C9	120.89 (17)		
			
C14—O2—C1—C2	169.13 (14)	C10—C11—C12—C13	0.0 (3)
C14—O2—C1—C6	−13.1 (2)	O1—C8—C13—C12	179.89 (14)
O2—C1—C2—C3	177.58 (15)	C9—C8—C13—C12	−1.9 (2)
C6—C1—C2—C3	−0.2 (2)	O1—C8—C13—C14	−2.2 (2)
C1—C2—C3—C4	−0.2 (3)	C9—C8—C13—C14	175.99 (15)
C2—C3—C4—C5	0.1 (3)	C11—C12—C13—C8	1.2 (2)
C3—C4—C5—C6	0.5 (3)	C11—C12—C13—C14	−176.69 (16)
C4—C5—C6—C1	−0.9 (2)	C15—N1—C14—O2	77.64 (18)
C4—C5—C6—C7	−177.79 (15)	C7—N1—C14—O2	−67.97 (16)
O2—C1—C6—C5	−176.91 (14)	C15—N1—C14—C13	−160.16 (14)
C2—C1—C6—C5	0.8 (2)	C7—N1—C14—C13	54.23 (17)
O2—C1—C6—C7	0.0 (2)	C1—O2—C14—N1	46.56 (17)
C2—C1—C6—C7	177.66 (14)	C1—O2—C14—C13	−73.93 (16)
C8—O1—C7—N1	46.98 (17)	C8—C13—C14—N1	−19.1 (2)
C8—O1—C7—C6	−73.99 (17)	C12—C13—C14—N1	158.72 (15)
C15—N1—C7—O1	143.14 (14)	C8—C13—C14—O2	103.18 (17)
C14—N1—C7—O1	−70.99 (16)	C12—C13—C14—O2	−79.01 (19)
C15—N1—C7—C6	−94.27 (17)	C18—N3—C15—N2	−0.5 (3)
C14—N1—C7—C6	51.60 (17)	C18—N3—C15—N1	177.54 (18)
C5—C6—C7—O1	−81.72 (18)	C16—N2—C15—N3	0.9 (3)
C1—C6—C7—O1	101.46 (17)	C16—N2—C15—N1	−177.13 (18)
C5—C6—C7—N1	157.40 (14)	C14—N1—C15—N3	21.1 (2)
C1—C6—C7—N1	−19.42 (19)	C7—N1—C15—N3	163.28 (15)
C7—O1—C8—C13	−11.9 (2)	C14—N1—C15—N2	−160.68 (15)
C7—O1—C8—C9	169.77 (14)	C7—N1—C15—N2	−18.5 (2)
O1—C8—C9—C10	179.70 (15)	C15—N2—C16—C17	−0.7 (4)
C13—C8—C9—C10	1.4 (3)	N2—C16—C17—C18	0.2 (4)
C8—C9—C10—C11	−0.2 (3)	C15—N3—C18—C17	−0.1 (4)
C9—C10—C11—C12	−0.5 (3)	C16—C17—C18—N3	0.3 (4)

### Hydrogen-bond geometry (Å, °)

**Table:**

*D*—H···*A*	*D*—H	H···*A*	*D*···*A*	*D*—H···*A*
C5—H5A···O2^i^	0.93	2.48	3.199 (2)	134

Symmetry codes: (i) *x*, *y*−1, *z*.
